# Effect of Treatment with Progestins and Antiplatelet Agents on IVF in Women with Adenomyosis and Recurrent Implantation Failure

**DOI:** 10.3390/diagnostics15010030

**Published:** 2024-12-26

**Authors:** Irina Pacu, Nikolaos Zygouropoulos, Giorgia Zampieri, Aida Petca, Mircea Octavian Poenaru, Cringu Antoniu Ionescu

**Affiliations:** 1 Department of Obstetrics & Gynecology, Faculty of Medicine, “Carol Davila” University of Medicine and Pharmacy, 020021 Bucharest, Romania; irinapacu@hotmail.com (I.P.);; 2 “St. Pantelimon” Emergency Clinical Hospital, 021623 Bucharest, Romania; 3 “Elias” Emergency University Hospital, 011461 Bucharest, Romania; 4 “Bucur” Maternity, Saint Ioan Emergency Clinical Hospital, 012363 Bucharest, Romania

**Keywords:** adenomyosis, recurrent implantation failure, junctional zone, uterine peristaltic wave

## Abstract

**Background:** This prospective study aims to identify the effect of the dienogest 2 mg/day and aspirin 150 mg/day combined treatment for two months before frozen ET on the assisted reproduction outcome in women with adenomyosis and recurrent implantation failure (RIF). **Methods:** Patients were selected based on specific criteria and divided into two groups (with and without treatment). Preimplantation biochemical parameters and ultrasonographic features (endometrial thickness, uterine peristalsis, and junctional zone thickness) were compared with pregnancy rate in a non-natural cycle frozen embryo transfer technique. A comparison between the two study groups indicated an increased successful implantation rate and clinical pregnancy rate (25% vs. 7.4%). **Results**: These results were attributed to the reduced uterine peristalsis and the reduction in thickness of the junctional zone following treatment. Available data were limited due to the nature of the study though maximal effort was exerted for the selected patients between groups to be as demographically similar and free from other potential pathology that may affect the results. **Conclusions:** In conclusion, it appears that the above stated treatment improves outcomes in women with adenomyosis and RIF; the parameters used may provide an insight as to the reasons why this occurs, though an explanation of the molecular mechanisms is still elusive.

## 1. Introduction

Over the past few decades, infertility has become a major women’s health issue. About 36% of infertility cases are attributed to women with endometriosis and adenomyosis, who often undergo in vitro fertilization (IVF) procedures [1]. During reproductive years, adenomyosis can be identified in about 35% of women, with or without fertility problems [2]. Adenomyosis represents the presence of ectopic, non-neoplastic endometrial glands, and stroma located at the myometrial level. Usually, the myometrial tissue undergoes hyperplasia and hypertrophy [1]. These deviations from the normal uterine structure are related to infertility and can usually only be treated with assisted reproduction techniques (ART). Women with adenomyosis who undergo assisted reproduction (AR) often face recurrent implantation failure (RIF) which results in a lower rate of pregnancies even when good quality embryos are available. While there are many efforts to improve the quality of embryos, very few are focused on improving endometrial quality in clinical practice. Our study aims to identify personalized therapies that can improve the chances of pregnancy in women with adenomyosis, infertility, and RIF.

### 1.1. Endometrial Receptivity in Adenomyosis

The quality of embryos and the receptivity of the endometrium are the primary factors that affect pregnancy rates in assisted reproduction (AR). The most crucial event is when a high-quality embryo finds an endometrium that is receptive to implantation. The process of decidualization in the endometrium is induced by the production of progesterone from the corpus luteum or, in the case of AR, through the use of exogenous progesterone [1]. There are many important molecular events that occur during the menstrual cycle, with the homebox (Hox) genes playing a vital role in encoding proteins. HOX A-10 and HOX A-11 are expressed in the endometrium during the proliferative and midsecretory phases, provided that there is a good progesterone environment [3,4]. During the secretory phase, there is a local increase in prostaglandins, as well as a range of growth factors, cytokines, hormones, adhesion molecules, and transcription factors [5,6,7].

There is a significant amount of evidence that suggests infertility in endometriosis is caused by alterations in molecular expression and suboptimal endometrial receptivity [1]. The expression of the HOX A-10 gene is reduced in women with adenomyosis [8]. Additionally, some inflammatory markers, such as IL-1beta, NK cells, and corticotropin-releasing hormone, are increased [9]. IL-11 plays a crucial role in regulating trophoblastic invasion, and adenomyosis is associated with decreased levels of IL-11, which can lead to early pregnancy loss. In adenomyosis, the local endometrial metabolism of estrogens is abnormal, and there is a relative local progesterone resistance due to the downregulation of progesterone receptors [10].

Uterine peristalsis plays a crucial role in the process of implantation and can be easily evaluated through ultrasound. The contractions of the uterus run from the cervix to the fundus in a non-pregnant uterus, while during pregnancy, the contractions run from the fundus to the cervix. With the advancements in AR, there have been numerous studies (such as Zhu et al., 2014 [11]) that have explored the significance of uterine peristalsis in successful implantation. It is well established that touching the fundus of the uterus with the catheter can cause a lot of peristaltic activity, which can harm implantation by expelling the fluid from the uterine cavity [11]. The inner myometrium that is closest to the endometrial cavity is the most involved structure in uterine peristalsis [1]. In adenomyosis, there is a thickening of a particular structure called the junctional zone (JZ). This zone is identified as a low-intensity band between the endometrium and myometrium in resonance imaging (MRI). The thickening of the JZ is due to the proliferation of inner myocytes, and JZ hyperplasia causes the penetration of endometrial glands into the myometrium. Contraction waves of the uterus originate at this level, and abnormal uterine peristalsis in adenomyosis can cause infertility by leading to abnormal uterotubal transportation of sperm from the uterine cavity to the ovary during ovulation. Increased uterine peristalsis of the inner endometrium in adenomyosis is also related to local levels of estrogens, which activate local aromatase and sulfatase.

The thickness of the junctional zone (JZ) is a topic of discussion, and the most precise measurement is provided by MRI. Typically, the JZ measures between 5 and 8 mm, but in cases of adenomyosis, it can extend to over 12 mm, which serves as a diagnostic criterion. If the JZ is greater than 10 mm, the rate of implantation failure in IVF treatment is 95.8%, regardless of the quality of embryos. On the other hand, the thinner the JZ, the higher the chances of successful implantation in AR.

### 1.2. Therapeutic Strategies for Improving Implantation Rate in AR and Adenomyosis

Progestins have been used for a long time to treat adenomyosis and endometriosis. In adenomyosis, progestins cause endometrial decidualization and amenorrhea, and have positive effects on the JZ [12]. Several studies have shown a reduction in the diffusion coefficient of the JZ after continuous treatment with progestins [13]. Progestins reduce the proliferation of estradiol-induced cells and increase p27 protein expression in endometrial gland cells [11,12].

The abnormal proliferation of cells in the inner layer of the myometrium and the increase in a type of cells called myofibroblasts in the junctional zone (JZ) usually occur after small injuries associated with increased hiperperistalsis and dehiscences in the JZ [14,15]. Platelets have an important role as they induce epithelial–mesenchymal transition and fibroblats to myofibroblats transdifferentiation [16]. The final pathway is fibrosis and smooth muscle metaplasia [17]. In fact, in adenomyosis, there are repeated wounds, repeated tissue injury and repair and platelets are involved in these processes. Antiplatelet therapy may have positive effects on treating adenomyosis and improving implantation rate and the success of assisted reproduction in women with adenomyosis and infertility. Antiplatelet therapy works by inhibiting the synthesis of thromboxane A2, thus decreasing the expression of some proteins involved in adenomyosis [12]. However, there is currently a lack of studies on the use of agents targeting platelets. Studies on mice have shown that thromboxane A2 synthesis inhibitors can suppress myometrial infiltration and reduce uterine contractile waves [17].

## 2. Materials and Methods

### 2.1. Study Design

The present study was a prospective analysis of 94 women who underwent assisted reproduction between 1 January 2022 and 1 January 2024. The study was conducted at two medical institutions: the Genesis Athens Infertility Center and the St. Pantelimon Emergency Hospital Bucharest, Obstetrics and Gynecology Hospital.

This study investigates the effect of dienogest 2 mg/day and aspirin 150 mg/day combined treatment for two months before frozen ET on the assisted reproduction outcome in women with adenomyosis and RIF. Patient selection was made based on the inclusion and exclusion criteria detailed below. Written informed consent was obtained from each patient before inclusion. The data collected in this study were analyzed and subsequently reported. The study was approved by the Ethics Comision of St. Pantelimon Emergency Hospital Bucharest, Romania (approval number: No.99, approval date: 20 November 2024).

To ensure the accuracy of our study, we only included patients who met the inclusion criteria and did not meet any of the exclusion criteria to avoid confounding variables that could affect uterine receptivity and embryo quality.

Inclusion criteria

- Diagnosed with adenomyosis sustained by clinical examination and transvaginal ultrasound evaluation (as per the Morphological Uterus Assessment -MUSA-features of adenomyosis) or magnetic resonance imaging.
- Diagnosed with RIF: at least three embryo transfers (ET) independent of method (fresh or frozen embryos, including oocyte donation) with two good quality (Grade B) day-3 cleavage stage embryos (day-3 good quality embryos defined by at least seven uniform cells, <26% fragmentation, no vacuoles, normal zona pellucida, uniform blastomeres, and no other apparent morphological abnormalities-ASEBIR consensus [18]).
- Underwent easy embryo transfer (smooth introduction of the catheter without touching the uterine fundus, no cervix tenaculum used, and on retraction of the catheter no blood was seen on it).
- ET of frozen embryos in an artificial cycle.

Exclusion criteria.

- Other uterine pathologies present (submucous fibroids, cervical polips, congenital uterine malformations)
- Prior surgical interventions at the level of the uterus, cervix, or annexes (miometrectomy, metroplasty for congenital abnormalities, etc.).
- Other medical pathology (Cushing’s syndrome, Celiac disease).

Following the withdrawal of one patient from the study and two patients who opted not to proceed with the ET, a total of 94 patients were included in the study. Out of these, 54 patients did not receive any treatment before ET (Group NT), while 40 patients received treatment for 2 months before ET (Group T).

### 2.2. Embryo Transfer

In preparation for the ET, 2 mg of estradiol valerate was administered three times a day for preparation of the endometrium. Prior to increasing the estradiol valerate dose, endometrial thickness was measured by TVS. Once endometrial thickness of more or equal to 7 mm was achieved, progesterone was administered intramuscularly in increasing doses (20 mg, 40 mg, and 60 mg per day) for three days until ET day.

On ET day, two high-quality embryos were transferred into the uterus with the help of abdominal ultrasound guidance. The embryo transfers were conducted by an experienced doctor who did not know about the frequency of uterine peristalsis, using the same catheter every time. Blood samples were taken on the day of embryo transfer to determine the levels of serum E2 and P. Pregnancy was confirmed clinically through TVS visualization of a gestational sac, 2–3 weeks after a positive pregnancy test (serum or urine β-HCG). Luteal support was given for 6 weeks after confirming intrauterine pregnancy or until a negative serum β-HCG measurement ruled out pregnancy by intravaginal administration of 800 mg progesterone.

### 2.3. Observation and Assessment of Uterine Peristalsis and Junctional Zone Measurement

About an hour before ET, a TVS was performed using a General Electric Voluson E10 machine and a 4–9 MHz endocavity probe IC5-9-D by a single examiner (I.P.). The patient was in a relaxed lithotomy position during the procedure, and the probe was gently inserted into the vagina to avoid cervical stimulation. The examiner scanned the mid-sagittal plane of the uterus and then fixed the probe to record a 5 min video of uterine peristalsis. Each contraction is seen like a wave going through the endometrial cavity. Two independent observers, I.P. and N.Z., who were unaware of the patient’s group at the time, analyzed the video recordings. The mean uterine peristaltic frequency was the mean from the results of each observer.

Considering all the data from the literature [1,8,11,17] we consider a low peristaltic activity under 3 waves/minute.

**Figure 1:**
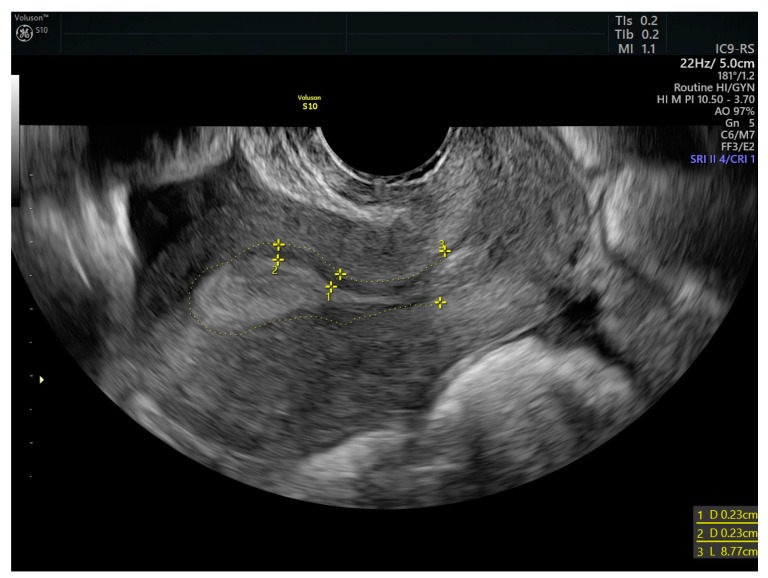
Sagital view of the uterus. Measurements 1 and 2 are measurements of JZ. Trace line 3 indicates separation between myometrium and subendometrium.

The measurement of the junctional zone (JZ) was performed using the same endocavity probe as the one used for uterine peristalsis. In a sagittal view at the end of the proliferative phase. There are three hyperechoic longitudinal lines separated by the hypoechogenic endometrium surrounded by the JZ appearing as a subendometrial hypoechoic line as shown in Figure 1 below.

### 2.4. Statistical Analysis

Statistical analysis was carried out using SPSS (IBM ver. 30.0.0.). Continuous data were presented as mean and standard deviation. Inter-observer agreement of uterine peristaltic wave frequency was evaluated with intraclass correlation coefficient. Continuous data were presented as mean and standard deviation (SD). A two-sample *t*-test was used to compare continuous variables between non-treatment and treatment groups. Categorical variables were compared by Chi-square test.

## 3. Results

In our study we have no difference between demographic characteristics or body mass index (BMI) of the two groups. Further, we do not have any significant differences between the partner’s age of the two groups. The mean age was 30.8 ± 2.6 versus 31.2 ± 2.9 years and the duration of infertility ranged from 1 to 12 years (mean 3.6 ± 0.7 versus 3.4 ± 0.8 years). In the nontreatment group, we have 13 cases with primary infertility and 11 cases in the second group, the group with therapy.

We have no difference between the two groups regarding the levels of basal FSH, LH, and estradiol, the level of estradiol on ET day, the endometrial thickness on ET day as well as rate of good quality embryos. A very small significant difference between the two groups was the level of serum progesterone on ET day with higher levels (53.3 ± 13.0 versus 60.7 ± 10.9 ng/mL, *p* value 0.046) (Table 1).

As shown in Table 2 we found a statistically significant difference between the two groups regarding mean uterine peristaltic wave frequency with a high activity for the non-treatment group 2.3 ± 1.0 waves/min comparing with a great improvement for the treatment group 1.6 ± 0.7 waves/min, *p* value 0.0118.

The difference in junctional zone thickness between the two groups has statistical significance (*p* value < 0.0001) with a mean value of 7.9 ± 1.4 mm in NT group versus 5.9 ± 1.1 mm in group T.

The higher the uterine peristaltic wave frequency is on ET day and the higher the JZ thickness value, the lower chance of pregnancy is with statistical significance difference between NT versus T group (*p* value 0.0001) (Table 2).

As we identified a statistically important correlation between uterine peristaltic wave frequency between the two groups (*p* = 0.0069, CI 95%) we tried to find the cut off value for predicting clinical pregnancy outcome, which was calculated to be approximately 1.6 waves/min. Of note is that, in other studies, a cut off of 2.45 waves/min as a cutoff peristaltic wave frequency for successful embryo transfer while frequencies below 2 waves/min where proven as optimal [11].

## 4. Discussion

Our present study is the first study which tries to find a personalized treatment for patients with adenomyosis and RIF. The treatment was cheap and easy, and the only inconvenience was the delay of the next frozen ET by two months. We combine two therapeutic strategies (the use of continuous progesterone therapy and antithroboxane A medication) for two months before ET to improve implantation rate by reducing uterine peristalsis and the thickness of JZ. All ultrasound measurements were carried out by a senior ultrasound doctor, and we used the same ultrasound instrument to be sure we have the standardization of measurements.

In both our study groups, we included patients with adenomyosis. The diagnosis was based on MRI or ultrasound criteria for adenomyosis. We consider TVS to be a first-line imaging method to diagnose this uterine pathology and all data from the literature reported both sensitivity and specificity of 78%, and the 3D exam is very useful for diagnosis and for a good evaluation of JZ [8]. For the inclusion criteria, we used MUSA ultrasound criteria as following [8], and we consider a positive diagnostic when having a minimum one of direct criteria or two indirect criteria:

- Direct criteria: myometrial cysts (anechoic content, low-level echogenity, ground-glass appearance, mixt echogenity), hyperechogenic islands (regular or irregular areas in the myometrium without any connection with endometrial cavity, as they represent ectopic endometrial tissue), hyperechogenic sub-endometrial lines that are perpendicular and continue to the uterine cavity.
- Indirect features: globular uterus, asymmetry between the thickness of anterior and posterior uterine wall with a difference more than 5 mm, hypo- and hyper-echogenic linear stripes in the uterine walls, trans-lesional vascularity (blood vessels perpendicular to the uterine cavity at Doppler exam), irregularity of JZ, interruption of JZ without consensus regarding the proportion if the interruption zones; the thickness of JZ is not included in the criteria of diagnosis and there is not a cut-off point of the thickness for the diagnostic.

The ultrasound evaluation of JZ is very accurate, and Reinhold et al. [19] reported that 2D-TVS and MRI have the same accuracy. Three-dimensional TVS is very useful in evaluating both JZ and the protrusion zones of endometrial tissue in the myometrium. There are a lot of studies to establish the influence of ultrasound manifestations in evaluation of success rate of IVF in adenomyosis and all of them concluded that a woman’s risk of infertility and the success rate of implantation in IVF depends directly with the increasing number of ultrasound signs of adenomyosis [18,20].

The therapeutic strategies we were using in our study were suggested by a lot of data from the literature that show that the only medical therapy which can be useful for the treatment of adenomyosis and for a greater chance for a successful pregnancy using IVF must be based on the pathogenetic mechanism of adenomyosis and infertility related to adenomyosis [1,11,12,17,20,21]. Understanding the role of JZ and uterine peristalsis, we identified a few types of medicines that have positive effect on these parameters. For the treatment of adenomyosis, a very important therapeutic and pathogenic line is HIFU, the use of GnRH analogs, oral contraceptives, a levonorgestrel-realeasing intrauterine device, or focused ultrasound surgery [12], but they are not useful when we are fighting with infertility.

To improve the rate of implantation for patients with RIF, we used the protocol we have previously described two months before frozen ET. In all cases, we have frozen embryos because we did not want to have high estrogen levels due to ovarian stimulation in the ET month and we wanted to be able to suppress the pathological aspects of adenomyosis with our protocol two months before ET. We used dienogest because it is well known it inhibits the proliferation of ectopic endometrial stromal tissue by arresting the cell cycle in G1/G0. A study on mice model of adenomyosis revealed that after using thromboxane, A2 synthesis inhibitors suppressed uterine hyperactivity and systemic corticosterone levels [17,20], but, until now, there are no studies regarding the effects of this therapy in adenomyosis in women.

Comparing the two study groups with adenomyosis and RIF, we found that our protocol is very useful to obtain a grater implantation rate and clinical pregnancy rate (25% vs. 7.4%) when used for a minimum of two months before ET progestin and antiplatelet therapy. These benefic results can be explained by reducing the frequency of uterine pulsatile waves and the thickness of JZ.

Regarding the width of the JZ, there is lot of evidence that increased JZ thickness is a poor prognostic factor for the embryo nidation, and it is associated with failure of implantation in IVF [12,21]. In adenomyosis, a high JZ thickness is considered to be an independent factor for RIF, and a JZ > 10 mm is associated with a rate of implantation failure of 95.8% [21]. Usually in adenomyosis, we found a JZ > 7 mm [12]; in our study without treatment, we have values of 7.9 ± 1.4 mm. After two months of treatment, we found a reduced JZ with mean values of 5.9 ± 1.1 mm and we consider it an important improvement of this factor related implantation failure (*p* value 0.0001). Many studies found that a reduced JZ is associated with a lower number of miscarriages or complications in the third trimester of pregnancy because of impaired of deep placentation [22,23]. We need further research to find out if our protocol has statistically significant improvements in the evolution of pregnancies with adenomyosis and IVF.

Regarding the uterine peristaltic waves we found (Table 3), a clinical pregnancy rate much higher Zhu et al. [11] and Fanchin et al. [24] suggest that this value can a be a good prognostic factor for implantation success [11]. With over 3 waves/min, we found only a few pregnancies. There are a lot of studies trying to improve pregnancy rates by using different agents to reduce uterine peristaltic waves [24,25,26,27,28]. Using progestins and antiplatelet agents two months before frozen ET seems to induce larger values of seric progesterone on ET day (Table 1), even though we used the same dose of exogenous progesterone the days before ET. Zhu et al. (2012) [11] found in some studies that the decreasing of supraphysiological serum progesterone can be responsible of the uptrend of uterine peristalsis [12]. In our study, we have only frozen ET and we have the preparation of the endometrium with estrogens and progesterone, but we cannot underestimate the possibility of an endogenous production of progesterone by the corpus luteum. Another limitation of the study is that we did not investigate the uterine peristalsis after ET with possible negative effects in embryo implantation. We also did not investigate the peristaltic wave types as the cervicofundal waves are different comparing with isthmic waves [12], but all these analyses are very complex and hard to be standardized.

## 5. Conclusions

According to all the literature data, we find a direct and statistically significant improvement of pregnancy rate for women with adenomyosis and RIF and AR by using a therapeutic protocol with progestins and antiplatelet agents two month before frozen ET. These benefits are due to reduction in the thickness of JZ and frequency of uterine waves which are mainly involved in the physiopathological aspects of implantation failure in these cases. The protocol is safe, cheap, and easily accepted by the patients. The limitation of the study is the limited number of patients and the need for further molecular research. The changes in JZ are very important in the treatment of female infertility not only related to endometriosis, and this protocol can be studied in infertility cases with no other etiopathogenical causes other than implantation failure, not only in AR.

## Figures and Tables

**Table 1:** Demographic and paraclinical comparison of non-treatment versus treatment group.

Characteristic	Group NT No Treatment (*n* = 47)	Group T Treatment (*n* = 40)	*p*-Value *
Age (years old)	30.8 ± 2.6	31.2 ± 2.9	0.6807
BMI (kg/m^2^)	23.5 ± 3.3	23.3 ± 3.3	0.746
Partner age (years old)	30.6 ± 2.4	30.5 ± 2.5	0.744
Duration of infertility (in years)	3.6 ± 0.7	3.4 ± 0.8	0.3029
Basal serum FSH (IU/L)	5.5 ± 2.2	5.9 ± 2.5	0.5822
Basal serum LH (IU/L)	5.4 ± 1.3	4.9 ± 1.5	0.1846
Basal serum E2 (pg/mL)	42.5 ± 11.8	38.2 ± 7.5	0.1608
Serum P on ET day (ng/mL)	53.3 ± 13.0	60.7 ± 10.9	**0.0461**
Serum E2 on ET day (pg/mL)	1032.1 ± 35.5	1038.1 ± 44.8	0.6112
Endometrium thickness on ET day (mm)	10.0 ± 1.3	9.6 ± 1.2	0.1071
Good quality embryo rate (%)	74.4 ± 6.2	75.1 ± 5.6	0.6721

* Two-sample *t*-test. In bold is the only *p*-value with statistical significance.

**Table 2:** Mean uterine peristalsis wave frequency on ET day, junctional zone thickness and clinical pregnancy rate in non-treatment versus treatment group.

Characteristic	Group NT No Treatment (*n* = 47)	Group T Treatment (*n* = 40)	*p*-Value	
Mean uterine peristaltic wave frequency (waves/min)	2.3 ± 1.0	1.6 ± 0.7	**0.0118**	*
Junctional zone thickness (mm)	7.9 ± 1.4	5.9 ± 1.1	**<0.0001**	*
Clinical pregnancy rate (%)	7.4	25.0	**0.0001**	**

* Two-sample *t*-test. ** Chi-square test. In bold is the only *p*-value with statistical significance.

**Table 3:** Comparison of uterine peristaltic wave frequency between clinically pregnant and non-pregnant women.

Uterine Peristaltic Wave Frequency [UPwf] (waves/min)	Non Pregnant (*n* = 40)	Clinically Pregnant (*n* = 7)	Pregnancy Rate by Upwf (%)
≤1.0	6	5	45.5
1.1–2.0	13	2	13.3
2.1–3.0	11	0	-
>3.0	10	0	-
**Mean**	2.2	1.0	*p*-value *
**Standard Deviation**	1.1	0.3	**0.0069**

* Two-sample *t*-test. In bold is the only *p*-value with statistical significance.

## Data Availability

All data (with exception actual experimental data) and information was collected through open or paid databases containing published journals. Experimental raw data collected during the study are available on request in accordance with the provisions stipulated in the patient consent for publication.
